# Effects of Substitution and Substrate Strain on the Structure and Properties of Orthorhombic Eu_1−*x*_Y*_x_*MnO_3_ (0 ≤ x ≤ 0.5) Thin Films

**DOI:** 10.3390/ma16134553

**Published:** 2023-06-23

**Authors:** Yonny Romaguera-Barcelay, Fábio Gabriel Figueiras, Ernesto Govea-Alcaide, Walter Ricardo Brito, Henrique Duarte da Fonseca Filho, Ariamna María Dip Gandarilla, Ştefan Ţălu, Pedro B. Tavares, Javier Pérez de la Cruz

**Affiliations:** 1 BioMark@UC, Faculty of Sciences and Technology, University of Coimbra, 3004-531 Coimbra, Portugal; 2 Department of Physics, Federal University of Amazonas, Manaus 69067-005, AM, Brazil; egoveaalcaide@gmail.com; 3 IFIMUP & Departamento de Física e Astronomia da Faculdade de Ciências da Universidade do Porto, 4169-007 Porto, Portugal; fabio.figueiras@fc.up.pt; 4 Laboratorio de Bioeletrônica e Eletroanalítica (LABEL), Department of Chemistry, Federal University of Amazonas, Manaus 69067-005, AM, Brazil; wrbrito@ufam.edu.br (W.R.B.); ariamna@ufam.edu.br (A.M.D.G.); 5 Laboratory of Synthesis of Nanomaterials and Nanoscopy, Physics Department, Federal University of Amazonas-UFAM, Manaus 69067-005, AM, Brazil; hdffilho@ufam.edu.br; 6 The Directorate of Research, Development and Innovation Management (DMCDI), The Technical University of Cluj-Napoca, 400020 Cluj-Napoca, Romania; 7 Centro de Química-Vila Real, Departamento de Química, ECVA, Universidade de Trás os Montes e Alto Douro, 5000-801 Vila Real, Portugal; ptavares@utad.pt; 8 ISQ-Instituto de Soldadura e Qualidade, 2740-120 Porto Salvo, Portugal; jpcruz@isq.pt

**Keywords:** Eu_1−*x*_Y*_x_*MnO_3_, manganites, spin-coating, thin films

## Abstract

**Highlights:**

- Orthorhombic Eu_1−*x*_Y*_x_*MnO_3_ (0.0 ≤ *x* ≤ 0.5) thin films were successfully synthesized by spin-coating.
- The film phase evidences an additional contribution to lattice strain with increasing Y-content.
- Appreciable modification of magnetic response from bulks to films due to structural distortions.

**Abstract:**

The effects on the structure and magnetic properties of Eu_1−*x*_Y*_x_*MnO_3_ (0.0 ≤ x ≤ 0.5) thin films due to lattice strain were investigated and compared with those obtained in equivalent composition ceramics. The films were deposited by spin-coating chemical solution onto Pt\TiO_2_\SiO_2_\Si (100) standard substrates. X-ray diffraction and Raman spectroscopy measurements revealed that all films crystallize in orthorhombic structure with space group *Pnma*, observing an added contraction of the unit cell with increasing Y-substitution ou Eu, corresponding to a broadening of the Mn-O1-Mn angle and a gradual decrease in magnetic order response.

## 1. Introduction

Transition metal oxides have many fascinating physical properties, such as high temperature, superconductivity, piezoelectricity, ferroelectricity, magnetism, and multiferroicity. Hence, these materials are still objects of intense scientific, engineering, and economic interest, considering the extensive technological applications already in use and further yet to be discovered. Rare-earth manganites *R*MnO_3_ are among some of the most relevant functional oxide materials, mainly due to intrinsic strong correlated electron mechanisms, leading to exhibiting structural-orbital-magnetic-electric coupling effects. In particular, it can be used for barriers and interfaces in controlled spin transport devices [1,2,3,4,5,6]. According to the ionic radius of rare earth elements, the manganites can exhibit two main distinct structural symmetries. The perovskite-like orthorhombic phase, comprising a network of octahedral MnO_6_ building blocks, surrounds relatively larger *R*^3+^ cations from La to Dy. In particular, systems like GdMnO_3_, TbMnO_3_, and DyMnO_3_ reveal magnetoelectric properties owed to competitive ferromagnetic (FM) and antiferromagnetic (AFM) exchanges. In such cases, the Mn-O1-Mn angle is considered the primary order parameter to understand the modulated spin-lattice coupling mediated ferroelectric (FE) ground states [7,8]. The hexagonal space group can be found in the *R*MnO_3_ systems of smaller *R*^3+^ cations, including Lu, Y, and Sc [9,10,11,12,13,14]. These compounds are based on MnO_5_ layers and present only AFM order at low temperatures due to weak spin-orbit coupling (<90 K). Nonetheless, the FE order prevails up to substantial high temperatures. In addition to ionic displacements in these systems, other complex mechanisms related to exchange effects of electron orbital polarization contribute to the ferroelectric state [15,16,17,18]. In this particular case, the Eu_1−*x*_Y*_x_*MnO_3_ system can exhibit both the orthorhombic (*x* ≤ 0.5) and hexagonal phase (*x* > 0.5), allowing the possibility of tuning the magnetic and polar phases through manipulation of the A-site size, the partial substitution of larger Eu^3+^ ions for smaller isovalent Y^3+^ ions without increasing the *R*-site magnetic complexity. The variation of the Mn-O1-Mn bond angle in these systems is also associated with the development of complex magnetic states and ferroelectric phases, leading to the magnetoelectric properties exhibited by this system [19,20,21]. The Eu_1−*x*_Y*_x_*MnO_3_ compound shows the characteristics of a paramagnetic to an incommensurate antiferromagnetic phase (AFM-1) at *Néel* temperature (T_N_), varying monotonously from 52−45 K (*x* = 0) to 45 K (*x* = 0.50) [7,22]. For *x* < 0.1, a transition from the AFM-1 to a canted A-type antiferromagnetic phase occurs at 43 K (*x* = 0) to 33 K (*x* = 0.1) [22]; next while it emerges for *x* < 0.25 [23]. For the compositions 0.25 < *x* < 0.35, a cycloidal modulated antiferromagnetic and ferroelectric phase (AFM-2) becomes apparent. For 0.35 < *x* < 0.55, a re-entrant magnetic phase, corresponding to two successive magnetic transitions, is associated with the cycloidal plane’s rotation from the ab to the bc plane [7,22,23]. This rotation implies that the spontaneous electric polarization changes from the *c*-axis to the *a*-axis [23]. Other authors consider, for 0.15 < *x* < 0.35, a further low temperature modulated conical magnetic and ferroelectric phase (AFM-3) [7,22,23,24]. However, the magnetic structure of low-temperature phases continues to be intensely studied. Besides the studies of Eu_1−*x*_Y*_x_*MnO_3_ as single crystals and conventional ceramics [22], it becomes relevant to investigate this system in the form of thin films since the properties may differ from those of bulk ceramics due to the influence of dimensional strain and the substrate interface. Even with their granular nature, thin films have attracted great interest and require a systematic comparison between the behavior of single crystals and ceramics [22,25,26]. In addition to contributing to extend the understanding and enhancing the underlying multiferroic mechanisms, thin film materials are crucial for developing potential applications and further fabrication of functional electro-optical devices. Chemical solution deposition methods like the sol-gel spin-coating technique provide higher composition control, lower processing temperatures, shorter fabrication time, and relatively low cost, compared to other chemical and physical vapor deposition thin-film-forming processes such as evaporation, pulsed laser, and sputtering [27].

As thin films can be subjected to strong elastic strains due to structural mismatch with respect to substrates, their intrinsic physical properties can be actively modified. These effects are under study on other strained perovskite thin films, which in bulk form exhibit orthorhombic structures: EuMnO_3_, GdMnO_3_, LaMnO_3_, DyMnO_3_, YMnO_3_, etc. [6,27]. From this perspective, the main objective of this work is to analyze and determine the effects of Y substitution of Eu and substrate strain on the structural and magnetic properties of orthorhombic Eu_1−*x*_Y*_x_*MnO_3_ thin films and, in particular, thin film oxides prepared by the sol-gel spin-coating method, taking into consideration some practical technical factors, including low production cost, equipment versatility, simplicity of preparation, composition control, and low sintering temperature. The experimental results encompassing crystal structure, dynamics, and the magnetic properties of these thin films are further compared with bulk ceramic samples of equivalent nominal composition (x = 0; 0.1; 0.2; 0.3; 0.4; 0.5).

## 2. Experimental Procedure

The studied Eu_1−*x*_Y*_x_*MnO_3_ thin films were prepared by a sol-gel method using standard Pt\TiO_2_\SiO_2_\Si (100) wafers as substrates. Details of precursor solutions and film preparation are available in refs. [27,28]. Thermogravimetric (TG) and differential thermogravimetric analyses (DTA) were conducted in the Eu_1−*x*_Y*_x_*MnO_3_ precursor solutions using a *Seteram Labsys* TG–DTA/DSC analyzer; each solution was previously dried at 100 °C for 48 h, forming the so-called dry solution. Afterward, it was heated in an air atmosphere at 10 °C/min from room temperature up to 1000 °C. The structural characterization of the films was carried out with a *PANalytical MRD* diffractometer, equipped with an *X’Celerator* detector in *Bragg-Brentano* geometry, using a Cu Kα radiation (*λ* = 1.5418 Å) source, a step of 0.017°/100 s from 20° up to 80° (2θ). Unpolarized micro-Raman spectroscopy was performed at ambient conditions for the 200–800 cm^−1^ spectral range, using a 514.5 nm Argon laser line as the excitation source. The incident power was kept below 10 mW to avoid local sample heating. The scattered light was analyzed by a *T64000 Jobin-Yvon* triple spectrometer, operating in triple subtractive mode and equipped with a liquid-nitrogen-cooled charge-coupled device. The spectral slit width was ∼1.5 cm^−1^. The parameters of the observed Raman modes (frequency, line width, and amplitude) were calculated using *IgorPro* software 6.0 from the best fit of a sum of damped oscillator functions [25]. Samples magnetization was measured using a *Quantum Design* SQUID magnetometer in reciprocating sample option (RSO) mode with a sensitivity of ∼10^−7^ emu. Measurements were carried out in a heating run from 5 to 300 K following protocols of zero field-cooled (ZFC) and field-cooled (FC) at 100 Oe. The experimental and calculated data were processed according to the error analysis procedures, accounting the intrinsic precision of the methods and corrections derived from the formulas used [29].

## 3. Results and Discussion

### 3.1. C.1. Phase Formation

**Figure 1:**
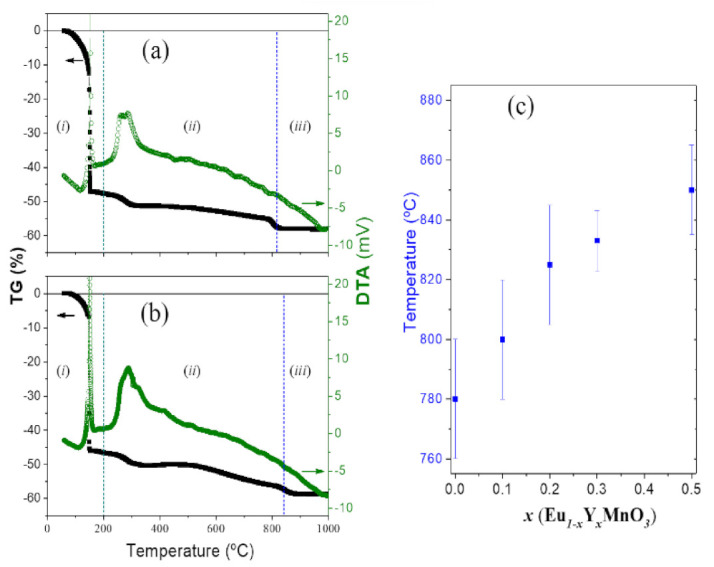
Thermal decomposition curves of the Eu_1−x_Y_x_MnO_3_ precursors solutions for *x* = (**a**) 0.2; and (**b**) 0.5. (**c**) Perovskite phase formation temperature for Eu_1−*x*_Y*_x_*MnO_3_ thin films.

Figure 1a,b shows the TG–DTA curves of the precursor solutions comprising the nominal concentrations of |Eu| = 1 − *x*; |Y| = *x*; in relation to |Mn| ≡ 1 cation.

The decomposition process includes three main steps: (*i*) solvent evaporation and (*ii*) calcination and pre-oxidation of precursor cations; these two preparation steps are similar to the ones reported for other oxide films [27,28]. The third step (*iii*) is the most important since it evolves the formation of the intended crystalline phase. The TG–DTA curves profiles, steps, and temperature intervals, obtained during the decomposition of each precursor solution exhibit slight variations depending on the ratio of substitution elements used. Overall, the formation of the Eu_1−*x*_Y*_x_*MnO_3_ perovskite crystalline phase is indicated by the precursor’s loss weight rate stabilizing beyond 770–880 °C. As seen in Figure 1c, the temperature threshold for the phase formation increases gradually with Y substitution (*x*) in relation to the EuMnO_3_ film. Consequently, the final sintering treatment was set at 800 °C for the *x* = 0 up to 850 °C for *x* = 0.5.

### 3.2. C.2. Surface Morphology

**Figure 2:**
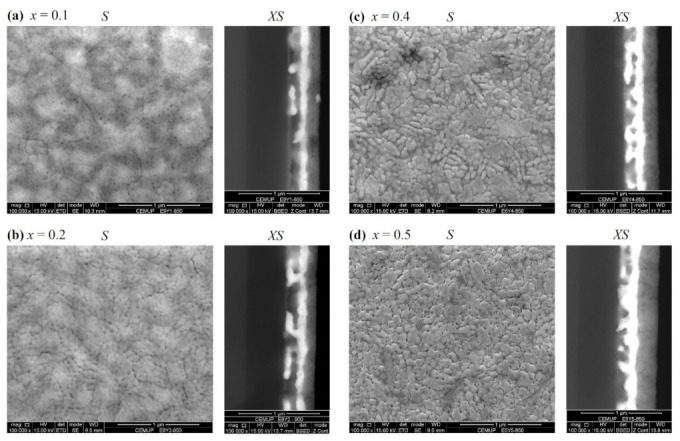
Eu_1−*x*_Y*_x_*MnO_3_ thin films representative SEM images obtained at ×10^5^ magnification, for, *x* = (**a**) 0.1; (**b**) 0.2; (**c**) 0.4; (**d**) 0.5, respective surface (left) and cross-section (right).

Representative SEM images of the Eu_1−*x*_Y*_x_*MnO_3_ films surface view and respective cross-section backscattering are shown in Figure 2a–d. Generally, each film exhibits a homogeneous crystallite size and a consistent dissemination of cracks throughout all surfaces.

As the content of Y^3+^ substitution increases, the average grain (crystallite) size and average area enclosed by cracks (agglomerates) decreases, whereas the film thickness gradually increases. A summary of the results extracted from these images is displayed in Table 1.

### 3.3. C.3. Crystal Structure

**Figure 3:**
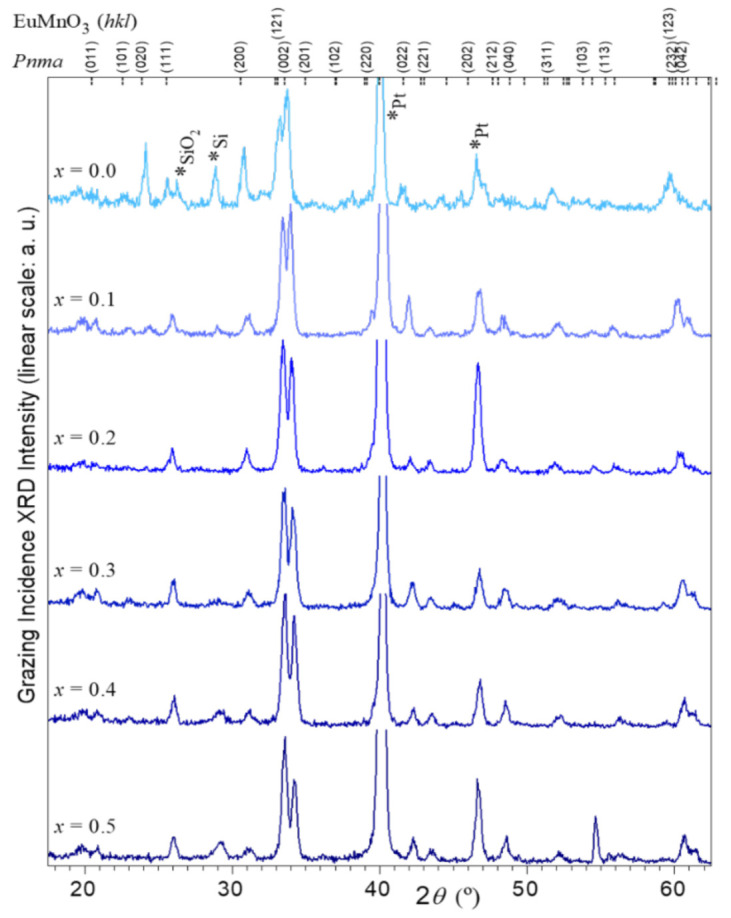
Spectra of X-ray diffraction on grazing angle of Eu_1−*x*_Y*_x_*MnO_3_ thin films with 0 ≤ *x* ≤ 0.5 concentration. The Bragg reflections belonging to the EuMnO_3_ (*x* = 0) phase are identified with Miller indexes. (*) Diffraction peaks of the substrate.

Figure 3 presents the grazing angle X-ray diffraction (GA-XRD) patterns obtained at ambient conditions from the series of Eu_1−*x*_Y*_x_*MnO_3_ thin films, where the diffraction peaks resultant from the substrate are labeled with (∗) symbols.

Thin films with composition 0 ≤ *x* ≤ 0.5 exhibit a typical XRD diffractogram profile associated with the *Pnma* (62) symmetry group, as observed in analogous bulk samples [22,25,26]. Likewise, no relevant secondary phases were detected. On the other hand, films with *x* ≥ 0.6 revealed concomitance of hexagonal and perovskite phases and are not the object of the present study since it is not viable to evaluate a direct correlation to each phase and its composition. A control film of YMnO_3_ (*x* = 1.0), synthesized by the same spin-coating sol-gel method and sintered at 850 °C, confirms the dominance of *P6_3_cm* symmetry [28,30,31]. Overall comparison of the graphs enables us to observe a shift of reflection peaks towards higher angles as *x* increases, suggesting a relative contraction of the unit cell volume. In the diffractogram of film EuMnO_3_ (*x* = 0.0) and to a lesser degree in film Eu_0.9_Y_0.1_MnO_3_ (*x* = 0.1), it is possible to discern that the intensity of the reflection peak at 2θ∼24°, indexed to planes (020) of orthorhombic phase, are over-proportional to the spectra expected from a random polycrystalline film. This specific feature, not detected on the other film diffractograms, is suggestive that the films with *x* = 0.0 and 0.1 can have partially grown with a preferred orientation along the *b*-axis. For x > 0.1, the films XRD do not show signs of oriented growth and are assumed to be dominated by polycrystalline form.

**Figure 4:**
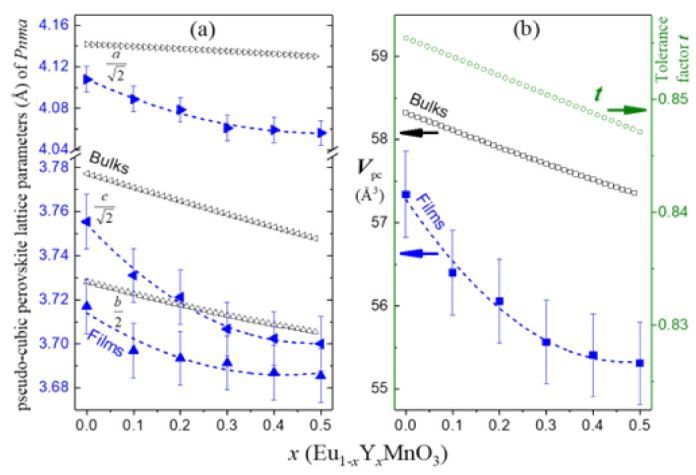
Dependence of the pseudo-cubic lattice parameters with *x* of the Eu_1−*x*_Y*_x_*MnO_3_ thin films (solid symbols) and ceramics [33] (respective open symbols). (**a**) (►) *a*_pc_, (▲) *b*_pc_, and (◄) *c*_pc_ where dashed lines correspond to respective quadratic degree polynomial function best fit to experimental results. (**b**) Calculated lattice volume ***V***_pc_ of (■) films, (□) bulks, and comparison to the theoretical tolerance factor (○) *t*.

Detailed (*Le Bail*) analysis of the diffractograms enables calculating the respective thin films lattice parameters. The fitting model used in the indexation to the *Pnma* space group and conventional *pseudo-Voigt* function to describe the peak shape [25]. Figure 4a plots the dependence of the pseudo-cubic (_pc_) lattice parameters defined as *a*_pc_ = *a*/√2, *b*_pc_ = *b*/2, and *c*_pc_ = *c*/√2 with *x*-composition, where *a*, *b*, and *c* are the conventional orthorhombic *Pnma* lattice parameters.

The relation *a*_pc_ > *c*_pc_ > *b*_pc_ is fulfilled for all compounds characteristic of the designated *O’* structure, typically found in rare-earth manganites. In this *O’* structure, the octahedral MnO_6_ frames are associated with a strong *Jahn-Teller* distortion and orbital ordering [32]. While in bulk ceramics, a linear decrease of lattice volume is observed in direct correlation with the cube of the effective A-site radius [25,26], it is evident that the series of films’ pseudo-cubic lattice parameters gradually decrease with increasing Y-substitution content in the range 0.0 to 0.5.

Table 2 resumes the values of quadratic |∆^2^*y*/∆*x*^2^| and linear |∆*y*/∆*x*| slopes obtained from best fitted quadratic degree polynomial functions (*y* = |∆^2^*y*/∆*x*^2^|·*x*^2^ + |∆*y*/∆*x*|·*x* + *y*_0_).

Analogously to Eu_1−*x*_Y*_x_*MnO_3_ ceramics, the lattice compression, due to Y^3+^ substitution, occurs in all axes, more manifested in *c-* and *b-* and less in *a-* directions. The quadratic terms |∆^2^*y*/∆*x*^2^| are residual, and the pseudo-cubic lattice parameters follow a straight linear slope with the relation |∆*a*/∆*x*| < |∆*b*/∆*x*| ≤ |∆*c*/∆*x*| [33], which can be traced directly to the system tolerance factor (*t* = <(1 − *x*)·*r*_Eu_ + *x*·*r*_Y_ + *r*_O_>/√2·<*r*_Mn_ + *r*_O_>). Quite differently from the described linear behavior reported for the ceramic series, the contraction of lattice parameters in the series of Eu_1−*x*_Y*_x_*MnO_3_ thin films follow a non-linear correlation to *t*. The results indicate an additional compression mechanism at play, patently due to the strain in the film phase induced by the substrate effect. Figure 4a,b expose the film lattice strain by separating it from the corresponding linear behavior found on bulks. The film lattice contractions are more relevant and evenly distributed in the basal plane *a-* and *c-* directions (< −0.5% to −1.7%) than in the *b*-direction (~−0.5%). This conjugation can explain why it is possible to advocate partial preferential growth of the film phase with *x* = 0 compatible with a less strained *b*-axis. On films, the relations become |∆*a*/∆*x*| ≈ |∆*c*/∆*x*| > |∆*b*/∆*x*|, respectively, ∼10, ∼3.5, and ∼2 times larger than reported for ceramics. Moreover, the quadratic terms have a similar magnitude to the linear ones and follow the same distribution |∆^2^*a*/∆*x*^2^| ≈ |∆*c*^2^/∆*x*^2^| > |∆*b*^2^/∆*x*^2^|. Subsequently, the continuous reduction of the unit cell parameters and volume attests that the systems remain in the original *Pnma* phase, suggesting a supplementary adjustment of MnO_6_ octahedra distortions and tilting [34]. The lattice volume contraction is near −1.7% for *x* = 0 and reaches a maximum of −3.7% for *x* ≥ 0.3.

### 3.4. C.4. Raman Spectroscopy

**Figure 5:**
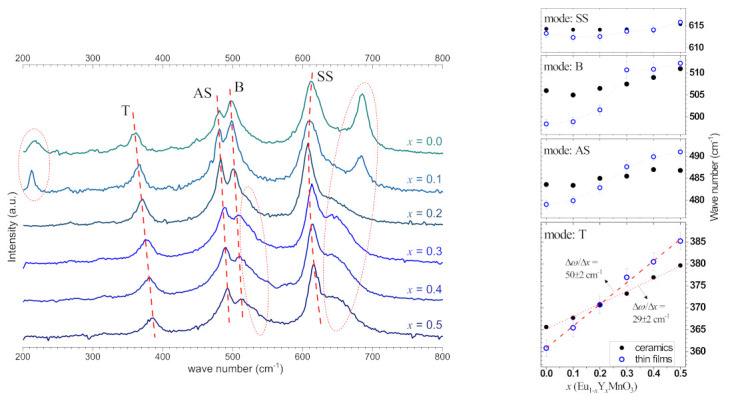
Un-polarized Raman spectra of Eu_1−*x*_Y*_x_*MnO_3_ for 0 ≤ *x* ≤ 0.5 thin films annealed at 850 °C. Mode assignment: T—tilt mode of the MnO_6_ octahedra (symmetry A_g_); SS—symmetric stretching mode (symmetry B_2g_); B—bending mode (symmetry B_2g_), and AS—*Jahn-Teller* type asymmetric stretching mode (symmetry A_g_). Dashed red lines are eyes-guides for the indicated modes identification. Insets: detailed graphs of the T; AS; B; and SS modes dependence with *x* in the Eu_1−*x*_Y*_x_*MnO_3_ thin films and ceramics [24].

Figure 5 shows the unpolarized Raman spectra obtained from the series of Eu_1−*x*_Y*_x_*MnO_3_ thin films recorded at ambient conditions.

Due to the main contribution of a polycrystalline phase in the series of samples, the experimental spectra simultaneously exhibited all Raman-active modes A_g_, B_1g_, B_2g,_ and B_3g_; the assignment [13,35,36] assumes the band at ∼359 cm^−1^ is resulting from the bending or tilt (T) mode of the MnO_6_ octahedra, which is activated by the (101) rotations (symmetry A_g_); the band at ∼478 cm^−1^ is assigned to a *Jahn-Teller* type asymmetric stretching (AS) mode also involving the O(II) atoms (symmetry A_g_); the band at ∼498 cm^−1^ is assigned to a bending mode (B) (symmetry B_2g_); and the band at ∼612 cm^−1^ is associated to a symmetric stretching mode (SS) involving the O(II) atoms (symmetry B_2g_). These modes can be detected in the range from 350 to 650 cm^−1^ and are also signaled in ceramic samples [25,35,36], corroborating the *Pnma* symmetry of the films already ascertained from the XRD data.

In the Raman spectrum of the Eu_1−*x*_Y*_x_*MnO_3,_ thin films with *x* = 0 and 0.1 are observed as additional modes at ∼220 and ∼685 cm^−1^; these can be interpreted as a Raman activation of active infrared modes – a direct consequence of the lattice distortions occurring in thin films due to the lattice mismatch between film and substrate and the film-substrate interaction [32], consistent with the partial presence of an orientated layer as mentioned from the XRD analysis. In samples with 0.2 ≤ x ≤ 0.5, the contribution of an orientated layer is not so significant. Hence, the mode at ∼220 cm^−1^ vanished, while the broad shoulder band above ∼620 cm^−1^ can still be attributed to distortions in the film lattice induced by the substrate effect. Still, due to the dominant polycrystalline structure, these distortions are disordered and relaxing through the film thickness, contributing to the convolution of peaks deriving from a distribution of modes.

Figure 5 shows the dependence in *x* of the peak center position wave number (*ω*) of the four most intense Raman modes assigned T, AS, B, and SS, measured in the films and compared to the ceramics with matching composition [25]. These modes are particularly interesting because they indicate the structural deformations in the Eu_1−x_Y_x_MnO_3_ thin films. For the thin films, the wave number of the tilt mode (T) increases linearly with Y^3+^ content with a slope ∆*ω*^T^/∆*x* films = 50 ± 2 cm^−1^, a similar linear behavior ∆*ω*^T^/∆*x* bulks = 29 ± 2 cm^−1^ is found for the ceramic’s samples [25]. Iliev et al. [35] demonstrated that such out-of-phase rotation mode and the tilt angle (*θ^T^*) exhibit a linear behavior with slope ∆*ω*^T^/∆*x* = 23.5 cm^−1^. By combining the above results, a variation of the rotation mode and the tilt angle with *x* can be obtained, for the series of Eu_1−*x*_Y*_x_*MnO_3_ thin films, of *∆θ^T^*/∆*x* = 2.1°. This value indicates that the volume reduction, anticipated from X-ray diffraction analysis, corresponds to an increase in the tilt angle with up to *x* = 0.5, close to 1°. While in Y-doped ceramics, the variation was ∆θ/∆*x* = 1.3°, representing an increase in the tilt angle of only 0.05° [25]. The symmetric stretching mode SS follows a near analogous constant (∆*ω*^SS^ ∼2 cm^−1^) behavior for films and ceramics, suggesting minor variations of Mn-O_2_ bond lengths with increasing *x*. However, in the same concentration range, the wave numbers measured for the thin films of the *Jahn-Teller* type asymmetric stretching mode (AS) and bending mode (B) exhibit a step-like discrepancy relative to the near linear ∆*ω*/∆*x* behavior identified in ceramics. In effect, specifically between *x* = 0.2 and 0.3, where the four mode defined curves intersect, the pseudo-perovskite lattice *c*_pc_ (films) and *b*_pc_ (bulks) lengths coincide, as can be seen in Figure 4a.

### 3.5. C.5. Magnetic Properties

**Figure 6:**
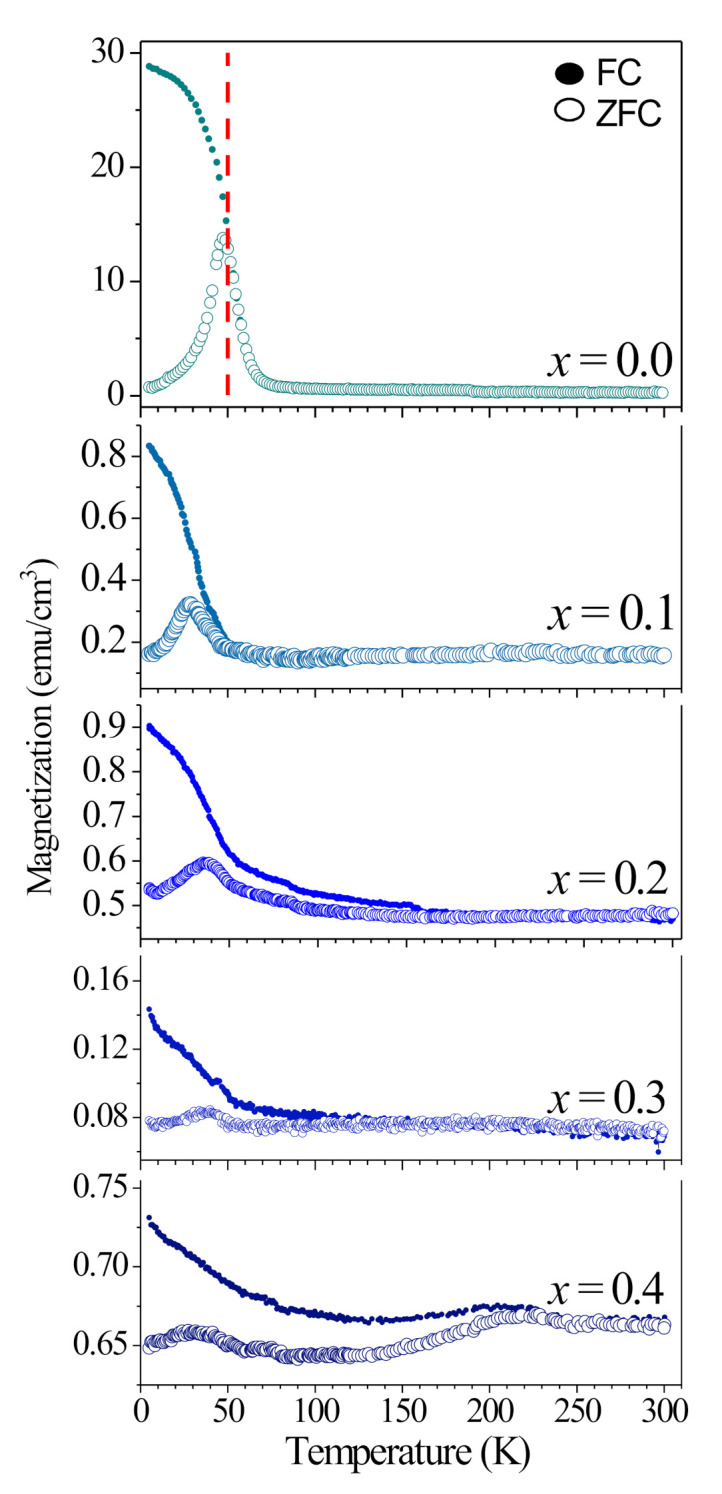
ZFC (open dots) and FC (closed dots) magnetization as a function of temperature for the Eu_1−*x*_Y*_x_*MnO_3_ thin films for 0 ≤ *x* ≤ 0.4.

The structural distortions associated with the film phase and Y^3+^ substitution are expected to significantly influence the magnetic properties of the studied samples, particularly the competitive ferromagnetic (FM) and antiferromagnetic (AFM) exchange interactions. Figure 6 presents the ZFC/FC magnetization measurements performed on the Eu_1−*x*_Y*_x_*MnO_3_ thin films from *x* = 0.0 to 0.4.

In overview, the experimental data follow a similar behavior to the ones reported in single crystal and ceramic samples of the equivalent compounds [22,25]. The difference between FC and ZFC magnetization ∆M (5 K) = M_FC_ (5 K)—M_ZFC_ (5 K) is 27.9 emu/cm^3^ for *x* = 0.0, but ensuing films 0.1 ≤ *x* ≤ 0.4 ∆M (5 K) yield, respectively, only 0.67; 0.36, 0.07; and 0.08 emu/cm^3^. In EuMnO_3_ and Eu_0.9_Y_0.1_MnO_3_ films, an expected irreversibility behavior is observed for temperatures up to T_N_ ∼50 K. Below this temperature, the characteristic weak FM arising from the canted A-type AFM was exhibited similar to EuMnO_3_ single crystals and ceramics samples [22,37]. However, in films with Y^3+^ concentrations ranging from 0.2 to 0.4, the irreversibility behavior persists at higher temperatures, making the precise determination of T_N_ difficult. The relative difference between FC and ZFC magnetization curves decreases with increasing Y^3+^ concentration, and the concavity of the FC curves inflect downward for *x* ≥ 0.3, as seen in Figure 6. It is reasonable to interpret the reduction of the induced magnetic moments in Eu_1−*x*_Y*_x_*MnO_3_ thin films with increasing *x* as a consequence of the volume contraction of the unit cell and the increase in the Mn-O1-Mn bond angle. While in films with *x* = 0.0 and 0.1, the weak FM stems from the canted A-type AFM, the incommensurate AFM for Y^3+^ concentration in the range from 0.2 to 0.4 may not be assessed as the cause of the irreversibility at temperatures above T_N_. In this case, the film-substrate interface may emulate an apparently emergent FM response caused by pinning local magnetic moments induced by the excitation magnetic field [38]. This weak FM character, prolonging above 100 K, hinders the application of a Curie-Weiss model [6]. The previous results agree with those reported in Lu-doped EuMnO_3_ thin films [17,39]; however, irreversibility behavior seems more marked in these samples.

## 4. Conclusions

Thin films of Eu_1−*x*_Y*_x_*MnO_3_ (0 ≤ *x* ≤ 0.5) were successfully prepared from the precursor solutions and deposited onto Pt\TiO_2_\SiO_2_\Si (100) substrates. The X-ray diffraction analysis reveals that the films stabilize in a single *Pnma* orthorhombic phase up to x = 0.5, while further substitution results in phases concomitance or full crystallization for *x* = 1.0 on *P6_3_cm* hexagonal structure. Compared to equivalent orthorhombic Eu_1−*x*_Y*_x_*MnO_3_ bulk ceramics, the thin films revealed an enhanced decrease in unit cell volume with increasing substitution concentration, and a further increase in the inclination of the MnO_6_ octahedra was ascertained by Raman spectroscopy. In contrast to ceramics samples, the films significantly smeared out the paramagnetic-canted A-type AFM transition. A weak ferromagnetic character was detected even above 100 K and attributed to the pinning of local magnetic moments at the film/substrate interface. These results corroborate the pertinence of the substrate-induced strain to modify and tune the distortions of the compound crystal lattice and ferroic properties.

## Figures and Tables

**Table 1:** Topographic parameters calculated from SEM images of Eu_1−*x*_Y*_x_*MnO_3_ thin films. <*grain*>: average grain size ± 10%; <*aggl.*>: agglomerate size ± 10%; <*t*>: thickness ± 10%.

*x* (%)	*<grain>* (nm)	*<aggl.>* (nm)	*<t>* (nm)
0.1	220	525	140
0.2	90	275	160
0.3	50	145	180
0.4	50	105	200
0.5	40	80	250

**Table 2:** Slope values (in Å) degree 2 polynomial fit of the lattice parameter for Eu_1−*x*_Y*_x_*MnO_3_ thin films. The values obtained for ceramic samples of the same compound are extracted from Ref. [24].

Sample	*a*		*b*		*c*		*V*	
*Type*	*∆a/∆x*	*∆^2^a/∆x^2^*	*∆b/∆x*	*∆^2^b/∆x^2^*	*∆c/∆x*	*∆^2^c/∆x^2^*	*∆V/∆x*	*∆^2^V/∆x^2^*
Ceramics [26]	−0.020 ^± 0.03^	0.007 ^± 0.06^	−0.058 ^± 0.003^	0.022 ^± 0.006^	−0.064 ^± 0.002^	0.008 ^± 0.004^	−2.169 ^± 0.45^	0.386 ^± 0.86^
Thin films series	−0.206 ^± 0.03^	0.204 ^± 0.05^	-0.134 ^± 0.03^	0.159 ^± 0.07^	−0.226 ^± 0.02^	0.236 ^± 0.04^	−8.330^± 0.88^	8.88 ^± 1.70^

## Data Availability

The data used to support the findings of this study are available from the corresponding author upon request.
